# Predictive value of triglyceride glucose index and systemic inflammation index for diabetic retinopathy in Type-2 diabetes

**DOI:** 10.12669/pjms.41.4.11374

**Published:** 2025-04

**Authors:** Jie Li, Yanling Li, Qiannan Qi, Na Chen, Yueling Zhang

**Affiliations:** 1 Jie Li, 2^nd^ Department of Ophthalmology, Baoding No. 1 Central Hospital, Baoding, Hebei, China; 2 Yanling Li, Department of Respiration and Critical Care, Affiliated Hospital of Hebei University, Baoding, Hebei, China; 3 Qiannan Qi, Department of Neurology, Affiliated Hospital of Hebei University, Baoding, Hebei, China; 4 Na Chen, 2^nd^ Department of Ophthalmology, Baoding No. 1 Central Hospital, Baoding, Hebei, China; 5 Yueling Zhang, 2^nd^ Department of Ophthalmology, Baoding No. 1 Central Hospital, Baoding, Hebei, China

**Keywords:** Diabetic retinopathy, Systemic immune inflammation index, Type-2 diabetes mellitus, Triglyceride glucose index

## Abstract

**Objective:**

To explore the predictive efficacy of the triglyceride glucose (TyG) index and systemic immune inflammation index (SII) levels for diabetic retinopathy (DR) in patients with Type-2 diabetes mellitus (T2DM).

**Methods:**

A retrospective study was conducted on 200 T2DM patients admitted to the Ophthalmology Department at Baoding No.1 Central Hospital from June 2023 to December 2023. The patients were divided into 80 none-DR, 120 with DR. General information, biochemical tests, TyG index, and SII were collected. Receiver operating characteristic (ROC) curve analysis was used to evaluate the predictive ability of TyG index and SII for DR. Binary logistic regression was employed to assess the relationship between DR occurrence and TyG index and SII.

**Results:**

The mean TyG index and SII levels were higher in the DR group compared to none-DR group (P<0.05). The ROC curve analysis indicated that the combination of TyG index and SII had the highest area under the curve (AUC) at 0.912, surpassing the AUC of TyG index and SII individually. Binary logistic regression analysis demonstrated that TyG index and SII were independent predictors of DR occurrence in diabetic patients (OR=1.745, 95%CI: 1.213-2.547, *P*<0.05; OR=1.347, 95%CI: 1.115-1.843, *P*<0.05, respectively).

**Conclusion:**

TyG index and SII levels may predict the occurrence of DR in T2DM patients, and the combination of these two indices offers the best predictive efficacy for DR.

## INTRODUCTION

Diabetes mellitus (DM) is a common metabolic disease, with epidemiological studies showing that the prevalence of diabetes in Chinese adults has risen to 11.2%.^1^ Diabetic retinopathy (DR) is a common micro-vascular complication of DM, with the prevalence of DR among Chinese DM patients ranking first in blindness among individuals over 40 years old.^2^ DR often presents insidiously, making early detection challenging. The clinical presentation of visual impairment often signifies disease progression to advanced stages, resulting in sub-optimal therapeutic outcomes and elevated risks of disability and mortality in DM patients.^3^ Therefore, early detection and effective management of DR are crucial in protecting the vision of individuals with DM. However, early stages of DR may not manifest obvious symptoms of vision impairment, resulting in a low percentage of proactive clinical assessments among DM patients.^4^

Recent studies have indicated that abnormalities in retinal and systemic lipid metabolism may be an important risk factors for the occurrence and progression of DR.^5^-^7^ Insulin resistance (IR) is the basis of Type-2 diabetes mellitus (T2DM), which can also lead to disturbances in blood sugar and lipid metabolism, further damaging the body’s tissues and organs.^8^ Research has revealed that IR is a significant factor in the development of DR and serves as an important indicator of the severity of the condition.^9^ The triglyceride glucose (TyG) index, which is the product of triglycerides and fasting blood sugar, is a simple, cost-effective, and reliable indicator of insulin resistance closely related to the progression and complications of T2DM.^10^ The systemic immune-inflammation index (SII) refers to the product of platelets and neutrophils divided by lymphocytes, serving as a novel indicator that comprehensively reflects the overall immune-inflammatory status of the body.^11^ In recent years, SII has emerged as a significant marker in the study of T2DM and its chronic complications.^12^ Its simplicity in acquisition, ease of use, low cost, and lack of economic restrictions make it a potential next-generation, user-friendly indicator for clinicians to understand the occurrence and progression of T2DM and its chronic complications. Currently, there have been research reports on the relationship between TyG, SII, and DR^13^-^16^, but it is still unclear whether they both are important factors in the occurrence of DR. Therefore, this study aims to preliminarily explore the correlation between TyG, SII, and DR, as well as evaluate the combined predictive value of the two for DR.

## METHODS

Patients were thoroughly informed about the study’s objectives and provided signed consent forms. Experimental Design: A cross-sectional design was implemented in this study, utilizing clinical data collected from June 2023 to December 2023 at Baoding No.1 Central Hospital. The study sample consisted of 200 patients diagnosed with T2DM.

### Ethical Approval:

The research conducted in this study adhered strictly to the principles outlined in the Helsinki Declaration and obtained approval from the Ethics Committee at Baoding No.1 Central Hospital (approval number: 202312, date April 11, 2023).

### Inclusion criteria:

Participants included individuals between the ages of 18 and 80, diagnosed with T2DM in accordance with the 1999 World Health Organization Diagnostic Standards for Diabetes.

### Exclusion criteria:

Individuals with acute diabetes complications, acute stress states, non-healing diabetic foot ulcers, severe cardiac, liver, or renal insufficiencies, cancer, recent infections, immune system disorders, hematological disorders, hyperthyroid eye disease, glaucoma, or any other conditions hindering cooperation during fundoscopic examinations were excluded from the study. All patients underwent a fundus examination and were categorized into two groups based on the DR classification standards set forth by the International Society of Ophthalmology in 2002: the non-DR group (n=80) and the DR group (n=120).

Data collection and laboratory analysis involved the collection of comprehensive demographic information, such as age, sex, diabetes duration, drinking, smoking, blood pressure measurements (systolic and diastolic), height, and weight. Additionally, a range of laboratory tests were conducted, including assessments for neutrophils (N), lymphocytes (L), platelets (PLT), glycated hemoglobin (HbA1c), fasting blood glucose (FBG), serum uric acid (SUA), albumin (Alb), serum creatinine (Scr), triglycerides (TG), C-reactive protein (CRP) and triglycerides (TG). Body mass index (BMI) was calculated by dividing weight by the square of height. The SII was determined by multiplying the platelet count by the neutrophil count and dividing the result by the lymphocyte count. The TyG index is calculated using the formula Ln [fasting glucose (mg/dL) × fasting triglycerides (mg/dL)/2].

### Statistical analysis:

The statistical analysis of the gathered data was carried out using SPSS 22.0 software. The data processing and analysis were conducted using R version 4.4.0 (released on April 24, 2024), in combination with Zstats 1.0 software (available at www.zstats.net). For measurement data that followed a normal distribution, the results were presented as mean ± standard deviation. Group comparisons were conducted using two independent samples t-tests. In cases where the measurement data did not conform to a normal distribution, the median and quantile spacing [M (P25%, P75%)] were used, and group comparisons were made using the Mann-Whitney U-test. Trend analysis was performed using Chi-square trend testing via Mantel-Haenszel analysis. Independent risk factors for DR in T2DM patients were identified using binary logistic regression analysis. The diagnostic value of TyG index and SII for DR in T2DM patients was evaluated using receiver operating characteristic (ROC) curve analysis, including the calculation of the area under the curve (AUC). Statistical significance was determined at a threshold of *P*<0.05 for all analyses.

## RESULTS

**Fig.1:**
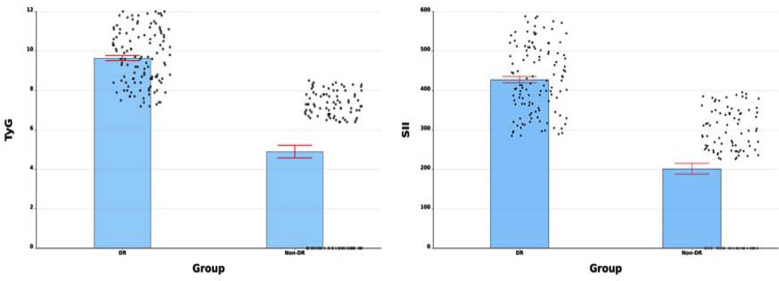
Comparing the SII index and TyG index between the two groups (*P* < 0.001 for both).

Among all these patients with T2DM, those with DR displayed significantly elevated levels of TyG and SII in comparison to non-DR patients (Fig.1, *P*<0.001). Furthermore, it was observed that DR patients had a notably longer duration of diabetes in contrast to non-DR patients (*P=*0.001, Table-I). Various laboratory parameters were found to be higher in the DR group, such as SBP, FBG, HbA1c%, CRP and neutrophil levels. However, no significant variances were noted between the DR and non-DR groups with regard to age, gender, smoking or drinking, DBP, BMI, Lymphocyte, PLT, Alb, Scr, SU and TG levels (Table-I, *P*>0.05).

**Table-I:** Comparison of general information between the two groups.

Variables	Non-DR (n = 80)	DR (n = 120)	P
Sex, n(%)			0.224
Male	47 (58.75)	60 (50.00)	
Drinking (n,%)	23 (28.75)	39 (32.50)	0.574
Smoking (n,%)	31 (38.75)	48 (40.00)	0.859
Diabetic Duration (Years, (M25, M75)	6.00 (6.00, 9.00)	9.00 (6.00, 14.00)	**0.001**
SBP (mmHg, Mean ± SD)	133.25 ± 6.65	141.75 ± 4.31	**<0.001**
DBP (mmHg, Mean ± SD)	85.99 ± 8.87	84.92 ± 8.54	0.397
BMI (Kg/m^2^, Mean ± SD)	26.97 ± 3.53	26.48 ± 3.19	0.317
SUA (mmoL/L, Mean ± SD)	467.61 ± 46.15	466.67 ± 46.30	0.888
TG (mmoL/L, Mean ± SD)	4.01 ± 0.99	3.74 ± 1.09	0.077
FBG (mmoL/L, Mean ± SD)	8.40 ± 2.19	9.04 ± 1.36	**0.021**
HbA1c% (Mean ± SD)	7.70 ± 0.45	8.00 ± 0.63	**<0.001**
CRP (mg/L, Mean ± SD)	7.78 ± 1.18	9.37 ± 0.95	**<0.001**
L (×10^9^/L,Mean ± SD)	1.70 ± 0.31	1.72 ± 0.32	0.631
N (×10^9^/L, Mean ± SD)	3.79 ± 0.62	5.53 ± 1.38	**<0.001**
PLT (×10^9^/L,Mean ± SD)	304.57 ± 74.11	290.13 ± 56.59	0.141
SII Index (×10^9^/L, Mean ± SD)	311.20 ± 52.37	434.61 ± 86.23	**<0.001**
TyG Index (Mean ± SD)	7.38 ± 0.63	9.58 ± 1.46	**<0.001**
Alb(g/L)	39.59 ± 3.03	39.71 ± 3.18	0.789
Scr (umol/L)	75.28 ± 12.03	78.15 ± 11.70	0.094

*Note:* Systolic Blood Pressure (SBP), Diastolic Blood Pressure (DBP), Body Mass Index (BMI), Serum Uric Acid (SUA), Triglycerides (TG), Fasting Blood Glucose (FBG), Glycated Hemoglobin (HbA1c), C-reactive protein (CRP), Lymphocytes (L), Neutrophils (N), Platelets (PLT), Systemic Immune-Inflammation (SII), Triglyceride Glucose (TyG), Albumin (Alb), Serum Creatinine (Scr).

Our study found that elevated levels of CRP, neutrophils, TyG index, and SII are all independent risk factors for DR (*P*<0.05, Table-II).

**Table-II:** Conducting a multiple factor analysis using binary logistic regression with diabetic retinopathy as the dependent variable.

Variables	P	OR (95%CI)
SBP (mmHg)	0.058	1.63 (0.91 ~ 2.65)
Diabetic duration(Years)	0.564	0.90 (0.62 ~ 1.29)
FBG (mmol/L)	0.421	1.32 (0.67 ~ 2.60)
HbA1c%	0.604	1.78 (0.20 ~ 15.49)
CRP (mg/L)	0.041	5.88 (1.07 ~ 32.30)
N (×10^9^/L)	0.024	14.00 (1.42 ~ 138.31)
SII Index (×10^9^/L)	0.008	1.03 (1.01 ~ 1.06)
TyG Index	0.019	8.49 (1.41 ~ 51.05)

*Note:* Systolic Blood Pressure (SBP), Fasting Blood Glucose (FBG), Glycated Hemoglobin (HbA1c), C-Reactive Protein (CRP), neutrophils (N), Systemic Immune-Inflammation (SII), Triglyceride Glucose (TyG).

**Fig.2:**
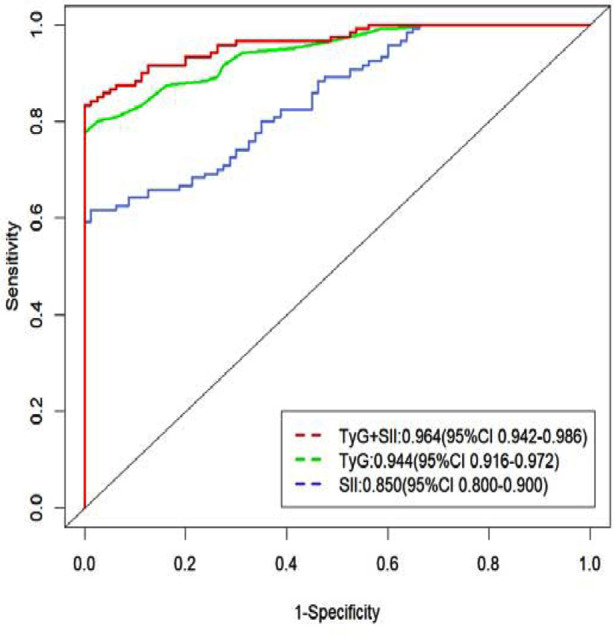
ROC curve analysis to assess the diagnostic accuracy of SII and TyG index in detecting DR in these patients.

As both the TyG index and SII were independent risk factors for DR, this study compared the two as predictive indicators for DR. The ROC curve showed that the optimal cutoff value for TyG was 8.25, with a sensitivity of 74.2% and specificity of 92.5%, and an AUC of 0.944. The optimal cutoff value for SII was 358.41, with a sensitivity of 77.5% and specificity of 85.0%, and an AUC of 0.850. When combined, the sensitivity of both indicators was 93.3% and specificity is 95.0%, with an AUC of 0.964. In conclusion, the synergistic utilization of both the TyG index and SII proved to be more effective in predicting DR compared to their individual application, thus demonstrating higher diagnostic efficacy. (Table-III, Fig.2).

**Table-III:** Comprehensive list of specific parameters for individual or combined ROC curves for SII index and TyG index.

Variable	AUC	95%Cl	Specificity	Sensitivity	Threshold
SII Index	0.850	0.800-0.900	0.85	0.775	358.41
TyG Index	0.944	0.916-0.972	0.93	0.742	8.25
Both	0.964	0.942-0.986	0.95	0.933	/

*Note:* Systemic Immune-Inflammation (SII), Triglyceride Glucose (TyG).

## DISCUSSION

This study found that TyG levels in DR patients were significantly higher than those non-DR patients, serving as an independent risk factor for the development of DR. While elevated blood glucose has traditionally been identified as the primary factor contributing to DR, studies have demonstrated that DR can still manifest or worsen in individuals with T2DM who maintain stable blood sugar and glycated hemoglobin levels.^17^ Studies have also indicated that early-stage DR is a reversible pathological change, underscoring the importance of early detection and effective management in preserving the vision of diabetic patients.^18^ Abnormal lipid metabolism has also been confirmed as a significant risk factor for the development of DR. Research has demonstrated that high blood lipids can accelerate glucose-induced mitochondrial damage, thereby hastening the onset and progression of DR.^19^ Studies have shown that DR patients have significantly elevated serum lipid levels compared to those without DR, and these levels are correlated with the severity of DR.^20^ The TyG index comprehensively reflects changes in fasting blood glucose and triglycerides in plasma, representing the combined action of glucose and lipids. Previous research has indicated that an elevated TyG index can predict the development of T2DM, the occurrence of diabetic nephropathy, the development of coronary artery stenosis, and the prognosis of patients with coronary artery disease.^21^ However, current studies on the correlation between the TyG index and DR have yielded conflicting conclusions. Nonetheless, most studies suggest that an elevated TyG index is positively correlated with the severity of DR, identifying it as a risk factor for DR and a predictor of its progression.^22^ The findings of this study align with the results of previous research mentioned above.

Our study found that SII levels were higher in the DR group compared to the non-DR group. In recent years, SII has emerged as a novel inflammatory marker. SII, which combines neutrophils, platelets, and lymphocytes, is a novel and easily accessible systemic marker of inflammation and immunity, providing a more comprehensive reflection of the balance of inflammation and immunity in the body compared to platelet-to-lymphocyte ratio and neutrophil-to-lymphocyte ratio.^23^ Neutrophils are the immune cells that first respond when inflammation occurs in the body, assisting in the aggregation of macrophages and interacting with antigen-presenting cells to further promote chronic inflammation. Platelets, known as “inflammatory cells,” play a crucial role by adhering to endothelial cells and white blood cells when activated, inducing an inflammatory response by releasing pro-inflammatory compounds. Lymphocytes, as a part of adaptive immunity, play an important role in innate immunity, acting as inflammation mediators with regulatory and protective functions. Studies have shown that SII encompasses platelets and a variety of inflammatory cells present in white blood cells, involving different immune regulatory pathways within the body.^24^ It is noteworthy that SII does not require active patient involvement, making it particularly valuable for evaluating diabetic patients with mobility limitations or cognitive impairments. Research has established a strong connection between SII and diabetes. SII can be utilized in predicting and assessing conditions such as diabetic nephropathy and depression.^25^,^26^ Our results confirmed that SII could serve as autonomous risk factors for evaluating the prevalence of DR in individuals with T2DM.

This study also revealed that both SII and TyG indices demonstrated strong predictive value for identifying the risk of DR in patients with T2DM. However, it is important to note that these biomarkers are not substitutes for the gold standard diagnostic methods, such as ophthalmoscopy and retinal imaging, which are essential for confirming the presence and severity of DR. Instead, SII and TyG may serve as useful adjunct tools for risk stratification and early identification of patients who may benefit from more frequent ophthalmologic evaluations.

### Limitations:

This study presents several limitations that should be addressed. Firstly, it was conducted as a single-center study with a limited sample size. The study did not incorporate healthy controls for comparison. Secondly, the study design was retrospective, suggesting a need for prospectively multicenter research to confirm the findings. Thirdly, during the patients’ hospitalization, SII and TyG were only tested once, lacking the assessment of their dynamic changes, suggesting the need for follow-up and enhancement in future studies. Furthermore, there were incomplete data on diabetes treatment, postprandial blood glucose levels, and retinal or choroidal parameters, necessitating further investigation. Additionally, the study only utilized a single blood test to obtain complete blood counts, which may not provide a comprehensive monitoring of changes in blood parameters over time. Lastly, the study did not include additional examinations of blood or eye tissues to analyze other inflammatory markers like cytokines and procalcitonin.

## CONCLUSIONS

Our study reveals that the average TyG and SII levels were notably elevated in the DR group when compared to the non-DR group. Furthermore, the simplicity of calculating and applying TyG and SII, along with their cost-effectiveness, make them valuable tools in identifying potential risk factors for DR in T2DM patients. Both SII and TyG are identified as independent risk factors for DR, with higher levels potentially indicating the presence of DR in T2DM patients.

### Authors’ Contributions:

**JL:** Study design, literature search and manuscript writing.

**YL and QQ:** Collected and analyzed clinical data. Critical Review.

**NC and YZ:** Formal analysis, literature review and revision.

**JL** is responsible and accountable for the accuracy or integrity of the work.

All authors have contributed to the study and approved the submitted version.
